# Clinical significance and risk factors of urethrovesical anastomotic urinary leakage following robot-assisted radical prostatectomy: a multi-institutional study

**DOI:** 10.1186/s12894-021-00844-1

**Published:** 2021-05-03

**Authors:** Shigenori Kakutani, Yuta Takeshima, Yuta Yamada, Tetsuya Fujimura, Shoichi Nagamoto, Yutaka Enomoto, Yuji Hakozaki, Naoki Kimura, Taro Teshima, Yoshiyuki Akiyama, Yusuke Sato, Taketo Kawai, Daisuke Yamada, Haruki Kume

**Affiliations:** 1 Department of Urology, Chiba Tokushukai Hospital, Chiba, Japan; 2 Department of Urology, Graduate School of Medicine, The University of Tokyo, 7-3-1 Hongo, Bunkyo-Ku, Tokyo, 113-8655 Japan; 3 Division of Innovative Cancer Therapy, Advanced Research Center, The Institute of Medical Science, The University of Tokyo, Minato-Ku, Tokyo, Japan; 4 Department of Urology, Jichi Medical University, Tochigi, Japan; 5 Department of Urology, Mitsui Memorial Hospital, Tokyo, Japan

**Keywords:** Anastomotic urinary leakage, Urethrovesical anastomosis, Urinary incontinence, Robot-assisted radical prostatectomy, RARP

## Abstract

**Background:**

There has been a limited number of reports on the significance and risk factors of urethrovesical anastomotic urinary leakage (AUL) following robot-assisted radical prostatectomy (RARP). We aimed to analyze the clinical significance of AUL and evaluated its risk factors.

**Methods:**

We conducted a multi-institutional study to review patients with prostate cancer undergoing RARP in three centers (The University of Tokyo Hospital, Mitsui Memorial Hospital, and Chiba Tokushukai Hospital). “Positive AUL” was defined as urinary extravasation at the anastomosis detected by post-operative cystogram and was further categorized into minor or major AUL. Univariate and multivariate analyses were performed to identify predictors of AUL. Postoperative continence rates and time to achieve continence were also analyzed.

**Results:**

A total of 942 patients underwent RARP for prostate cancer in 3 centers. Of these patients, a cystogram after the RARP procedure was not performed in 26 patients leaving 916 patients for the final analysis. AUL was observed in 56 patients (6.1%); 34 patients (3.7%) with minor AUL and 22 patients (2.4%) with major AUL. Patients with major AUL exhibited a significantly longer time to achieve continence than those without major AUL. Multivariate analysis demonstrated that longer console time (≥ 184 min) was significantly associated with overall AUL, and higher body mass index (≥ 25 g/kg^2^) was a significant predictor of both major and overall AUL.

**Conclusions:**

The presence of major AUL was associated with the achievement of urinary continence, suggesting clinical relevance of its diagnosis by postoperative cystogram. A selective cystogram has been proposed for high-risk cases. Furthermore, identification of the risk factors of AUL will lead to optimal application.

## Background

Robot-assisted radical prostatectomy (RARP) has become the most utilized treatment modality for localized prostate cancer in the past two decades [1, 2]. The improved vision of pelvic anatomy and the dexterity of robot-assisted surgical arms provided by RARP have allowed more precise and watertight urethrovesical anastomosis (UVA). A correlation between surgeon experience and quality of anastomosis has been reported, suggesting its utility as a benchmark of surgical skill for RARP [3]. This, in turn, has naturally led many to suggest that the improved quality of anastomosis resulted in reducing the incidence of urethrovesical anastomotic urinary leakage (AUL) following RARP as an advantage over conventional open retropubic radical prostatectomy (RRP) [4, 5]. However, there is presently little evidence to corroborate this, perhaps owing to a low number of events as in one study detecting no difference in AUL incidence between RRP and RARP (2.9% vs. 3.2%, *P* value = 0.49) [6]. In fact, we found that there is a lack of consensus on the importance of AUL in general, concerning its effect on the postoperative outcome, and its risk factors. A major factor contributing to this is that postoperative cystograms to detect AUL are not routinely taken at many centers around the world, leading to a significant variation in the definition of AUL, with some groups using findings of postoperative cystogram and others using confirmation of urine in the drain [7]. In 2008, Williams et al. proposed a systematic definition of AUL detected by cystogram and clarified its classification depending on severity and necessity of intervention, for objective quantification of AUL in future studies [8]. In this study, we conducted a routine postoperative cystogram following RARP and utilized this classification of cystographic findings to examine AUL objectively. Recognizing the factors associated with AUL is clinically important, since it may avert routine cystogram and provide beneficial information to surgeons for determination on patient selection regarding post-operative cystogram. To our knowledge, there is only one study that had presented statistically significant factors of AUL by performing a multivariate analysis in patients undergoing RARP [9]. Moreover, this is the first multi-institutional study to investigate the effect of AUL after RARP and its risk factors.

## Methods

### Patient population

The study was approved by the ethical committee of The University of Tokyo Hospital (ID: 2020039NI), the ethical committee of Mitsui Memorial hospital (ID: 2020C30), and the committee of Chiba Tokushukai Hospital (ID: 184). All clinical data were prospectively collected in a customized database and retrospectively analyzed. RARP was performed by multiple surgeons using da Vinci®Si or Xi Surgical Systems (Intuitive Surgical, Sunnydale, CA, USA) by a transperitoneal approach in 671 patients from Nov 2011 to May 2019 at The University of Tokyo Hospital, in 164 patients on the Si System from May 2017 to May 2019 at Chiba Tokushukai Hospital, and in 107 patients on the Xi System from Nov 2017 to May 2019 at Mitsui Memorial Hospital. Patients with non-metastatic prostate cancer with or without neoadjuvant hormonal therapy were treated with RARP. Contraindication of RARP included a history of rectal surgery or the presence of severe comorbidities.

### Surgical technique

RARP was performed and carried out in the same fashion in all 3 centers as previously described; UVA was completed after Rocco’s posterior rhabdosphincter reconstruction by a continuous running closure using two 3/0 absorbable monofilaments tied end-to-end [10, 11]. The integrity of UVA was routinely checked by an intraoperative flush test using 50–150 mL saline through the catheter. An additional suture was performed when needed to attain watertight anastomosis. A Foley catheter was placed into the bladder and the balloon was filled with 10 mL of water.

### Post-surgical examination

At 6–8 days after RARP, a cystogram of anteroposterior and oblique view was taken before removal of the urethral catheter by instilling 40–120 mL of saline-diluted iodinated contrast medium into the bladder. Positive AUL was defined as the ‘presence of urinary extravasation determined by cystogram’. In the absence of AUL, we removed the urethral catheter immediately or the next morning. Positive AUL was reviewed and subclassified into two categories; minor AUL within 6 cm of the UVA, in accordance with ‘grade 1 urethrovesical leak’, and major AUL beyond 6 cm from the UVA, in accordance with ‘grades 2 and 3 urethrovesical leaks’ [8]. In patients with minor AUL, the catheter was removed within a day after the cystogram. In patients with major AUL, the catheter was placed in situ until leakage was either undetectable on a repeat cystogram or recovered to minor AUL. Time to recovery of the urinary continence was calculated from the removal of the urethral catheter.

### Statistical evaluation

The collected clinical data were analyzed by using JMP Pro 15.1 software (SAS Institute, Cary, NC, USA). Valuables such as age, body mass index (BMI), initial PSA value, prostate volume, console time, blood loss volume, surgeon experience (≥ 20, 40, or 100 cases), cavernous nerve preservation status, and pathological findings (pGS ≥ 8, pT3 ≥ , positive surgical margin) were collected. Univariate and multivariate analyses using logistic regression analysis were performed to identify factors predicting AUL. Parameters that were significantly associated with AUL in the univariate analysis and known factors based on the previous reports were included in the multivariate analyses [12]. Kaplan–Meier curves were drawn, and the Wilcoxon test was used to analyze the time to recovery of continence (≤ one pad per day) after successful removal of the catheter. Cut-off values were basically determined by the median values of each parameter, except for BMI and prostate weight which were rounded to a clinically useful number. All tests were two-sided and a *P* value of < 0.05 was regarded as statistically significant.

## Results

Patient characteristics and perioperative status are shown in Table 1. A total of 942 patients underwent RARP procedure for prostate cancer in 3 centers (The University of Tokyo Hospital, Chiba Tokushukai Hospital, and Mitsui Memorial Hospital). The records of cystogram after RARP procedure was not available in 26 patients. A total of 916 patients were investigated for the final analysis. The number of patients included in this study was 645 patients for The University of Tokyo Hospital, 164 for Chiba Tokushukai Hospital, and 107 for Mitsui Memorial Hospital. 860 patients (93.9%) and 56 patients (6.1%) had negative and positive AUL, respectively. Of the patients with positive AUL, 34 (60.7%) were classified as minor AUL and 22 (39.3%) as major AUL. The median duration of catheterization was 7 days (IQR 6–8).

**Table 1:** Patients demographics

	Leakage (+)			Leakage (−)	Total
	Minor	Major	Minor + major		
*Anastomotic urinary leakage after RARP*					
Number of patients (%)	34 (3.7)	22 (2.4)	56 (6.1)	860 (93.9)	916
Age, (years)	68.5 (64–72)	65 (58–70)	68 (63–72)	68 (64–73)	68 (64–73)
BMI (kg/m^2^)	24.1 (22.2–26.5)	25.6 (24.4–28.1)	24.9 (22.9–26.7)	23.8 (21.9–25.6)	23.9 (22–25.6)
PSA (ng/mL)	8.8 (6.2–12.6)	8.3 (5.9–15.3)	8.5 (6.2–12.9)	7.6 (5.5–11.0)	7.6 (5.6–11.1)
Operation duration (min)	279 (203–341)	295 (232–379)	289 (215–349)	240 (195–283)	241 (196–287)
Console duration (min)	212.5 (155–266)	233 (168–322)	221.5 (164–271)	183 (143–221)	184 (145–226)
Prostate weight (g)	43.1 (34–70)	50.5 (40–74)	46.5 (38–72)	40.5 (32–52)	41 (32–53)
Blood loss volume (mL)	300 (100–725)	352 (174–666)	326 (150–700)	221 (100–442)	230 (100–450)
pGS ≥ 8 (%)	29.4	22.7	26.8	28.2	29.8
pT stage ≥ 3 (%)	23.5	40.9	30.4	30.6	30.6
PSM (%)	17.6	18.2	17.9	21.3	21.7

Median values are shown (interquartile range)

RARP, robot-assisted radical prostatectomy; BMI, body mass index; PSA, prostate specific antigen; pGS, pathological Gleason score; PSM, positive surgical margin

**Fig. 1:**
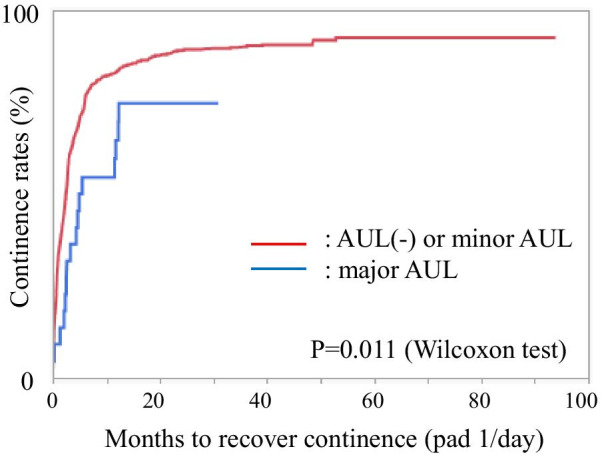
Months to recover continence in patients with or without major anastomotic urinary leakage. Kaplan–Meier method analysis revealed that there was a significant delay in days to recover continence less than one pad per day in patients with major AUL

Time to recovery of continence (≤ 1 pad/day) was significantly longer in patients with major AUL than those without major AUL (Fig. 1).

In the univariate analysis, BMI (≥ 25 kg/m^2^) and console time (≥ 184 min) were significantly associated with overall AUL (Table 2; *P* = 0.03 and *P* = 0.04, respectively). In the multivariate analysis, console time remained as the significant predictor of overall AUL. A similar analysis was conducted for major AUL (Table 3). In the univariate analysis, BMI (≥ 25 kg/m^2^) and console time (≥ 184 min.) were associated with major AUL (Table 3; *P* < 0.01 and *P* = 0.04, respectively). There was a tendency of observing more major and overall AUL in patients with prostate weight > 40 g (Tables 2, 3; *P* = 0.08, *P* = 0.006, respectively). In the multivariate analysis, BMI remained a significant predictor of major AUL. There was no significant association between surgeon experience and overall/major AUL.

**Table 2:** Univariate and multivariate analysis to predict overall anastomotic urinary leakage (AUL)

Variable	Cutoff	Univariate		Multivariate	
		Odds ratio (95%CI)	*P* value	Odds ratio (95%CI)	*P* value
Age, years	≥ 68 versus < 68	0.89 (0.52–1.54)	0.78		
BMI, kg/m^2^	≥ 25 versus < 25	2.02 (1.17–3.48)	0.01*	1.80 (1.03–3.12)	0.03*
iPSA, ng/mL	≥ 7.6 versus < 7.6	1.43 (0.82–2.48)	0.22		
Diabetes	With versus without	0.19 (0.04–0.80)	0.01*		
COPD	With versus without	0.45 (0.10–1.91)	0.42		
IHD	With versus without	1.55 (0.53–4.51)	0.34		
Abdominal surgery	With versus without	1.04 (0.56–1.92)	0.87		
Surgeon volume, cases	≥ 20 versus < 20	0.95 (0.55–1.66)	0.88		
	≥ 40 versus < 40	0.84 (0.48–1.49)	0.67		
	≥ 100 versus < 100	0.66 (0.27–1.57)	0.44		
Nerve sparing	With versus without	0.92 (0.52–1.64)	0.88		
Duration of console, min	≥ 184 versus < 184	2.00 (1.13–3.54)	0.01*	1.84 (1.03–3.28)	0.03*
Prostate weight, g	≥ 40 versus < 40	1.80 (0.99–3.27)	0.05	1.69 (0.93–3.09)	0.08
Blood, mL	≥ 230 versus < 230	1.26 (0.73–2.17)	0.41		
pGS ≥ 8	≥ versus <	0.86 (0.46–1.58)	0.76		
pT3	≥ versus <	0.98 (0.54–1.77)	1.00		
PSM	With versus without	0.80 (0.39–1.62)	0.61		

BMI, body mass index; COPD, chronic obstructive pulmonary disease; IHD, ischemic heart disease; PSA, prostate specific antigen; pGS, pathological Gleason score; PSM, positive surgical margin

Statistical significance is shown as * for *P* < 0.05

**Table 3:** Univariate and multivariate analysis to predict major anastomotic urinary leakage (AUL)

Variable	Cutoff	Univariate		Multivariate	
		Odds ratio (95%CI)	*P* value	Odds ratio (95%CI)	*P* value
Age, years	≥ 68 versus < 68	0.64 (0.27–1.50)	0.38		
BMI, kg/m^2^	≥ 25 versus < 25	4.30 (1.73–10.66)	< 0.01**	3.62 (1.44–9.09)	0.01*
iPSA, ng/mL	≥ 7.6 versus < 7.6	1.42 (0.60–3.36)	0.51		
Diabetes	With versus without	0.26 (0.03–1.95)	0.23		
COPD	With versus without	0	0.39		
IHD	With versus without	1.99 (0.45–8.83)	0.29		
Abdominal surgery	With versus without	0.44 (0.12–1.51)	0.22		
Surgeon volume, cases	≥ 20 versus < 20	0.96 (0.40–2.28)	1.00		
	≥ 40 versus < 40	0.44 (0.16–1.22)	0.12		
	≥ 100 versus < 100	0.88 (0.25–3.03)	1.00		
Nerve sparing	With versus without	1.03 (0.43–2.50)	1.00		
Duration of console, min	≥ 184 versus < 184	2.69 (1.04–6.94)	0.04*	2.23 (0.85–5.85)	0.09
Prostate weight, g	≥ 40 versus < 40	3.20 (1.07–9.56)	0.02*	2.47 (0.95–6.43)	0.06
Blood, mL	≥ 230 versus < 230	1.46 (0.61–3.46)	0.39		
pGS ≥ 8	≥ versus <	0.69 (0.25–1.90)	0.63		
pT3	≥ versus <	1.58 (0.67–3.76)	0.34		
PSM	With versus without	0.82 (0.27–2.47)	1.00		

BMI, body mass index; COPD, chronic obstructive pulmonary disease; IHD, ischemic heart disease; PSA, prostate specific antigen; pGS, pathological Gleason score; PSM, positive surgical margin

Statistical significance is shown as * for *P* < 0.05 and ** for *P* < 0.01

## Discussion

AUL has been reported to occur in 0.3–15.4% of patients after RARP [12]. In our study, the incidence of AUL detected by cytogram was 6.1%, which was compatible with other reports. However, the incidence of AUL varies greatly with parameters such as postoperative date, as demonstrated in a study that showed decreasing rates of AUL incidence with a lapse of time after RRP [13]. Although the timing of postoperative cystograms from past studies range from 3–18 days and requires standardization [14–16], we set the timing of cystogram at POD 6–8, which appears to be consistent with many of the previous reports [3, 5, 8, 9, 17].

We examined post-operative continence rates and found that time to recovery of continence was negatively affected by the presence of major AUL on cystogram. This result was in line with past reports that also found an association between AUL and post-operative continence [5, 18]. This leads us to believe that major AUL is of clinical importance and that examination of factors contributing to its incidence would be beneficial in predicting postoperative outcomes.

The predictive factors of AUL have been examined in multiple previous studies. Some factors presented were obesity/BMI, ischemic heart disease, urinary tract infection, excessive blood loss, surgeon experience, eversion of the bladder mucosa, prostate size, previous prostatic surgery, or details regarding surgical technique (bladder neck eversion, posterior reconstruction, etc.) [4, 9, 19–22]. In the univariate analysis of the present study, longer console time, larger prostate weight, and higher BMI was associated with AUL. Furthermore, in the multivariate analysis, longer console time and higher BMI were significant factors of overall AUL and higher BMI was a significant factor of major AUL.

Obesity may create difficulties in accessing the vesicourethral anastomosis site or to decrease visibility. Some reports state that this only applies to RRP, while others assert that obesity also affects outcomes of RARP negatively [21, 23]. Excessive bleeding may cause natural ischemia in the bladder or urethral tissue. They also lead to impairment of visibility, making precise suture placement difficult. Unavoidable suture for maintaining hemostasis may cause further ischemia. Past studies have also reported that the postoperative bleeding was a predictor of AUL and that this was likely the result of para-anastomotic hematoma delaying the healing of UVA [17, 24]. Tillier et al. asserted that men with larger prostate size and preoperative voiding problems were predictive of major leakage in RARP due to bladder neck reconstruction and the alignment of the bladder and urethra [9]. It is obvious that the larger the prostate, the longer the distance between the bladder neck and urethra after excision of the prostate, resulting in a more challenging UVA. Console time was not mentioned in any previous study, yet it remained a predictive factor in multivariate analysis for overall AUL in our study. We surmised that console time may have been a manifestation of the level of difficulty in conducting the surgery, and in some cases the difficulty of UVA.

The above observations seem to show that difficulty in conducting the anastomosis may be a predictor of AUL. Although based on one surgeon’s subjective assessment, Gnanapragasam et al. reported that a ‘difficult anastomosis’ was indeed an independent predictor of AUL [20]. There is also evidence that the quality of anastomosis was associated with surgeon experience [3]. We hypothesized that surgeon experience may be related to AUL and included surgeon experience as a factor, but we did not identify any association with the surgeon volume and AUL in our cohort. This may partly be due to the unique mentoring system we implemented, in which RARP was divided into sections, each with a designated time limit and blood loss criteria, and an experienced surgeon replaced an inexperienced surgeon for the remainder of the section upon exceeding the designated time or blood loss [10, 25].

A postoperative cystogram is considered a valuable diagnostic tool to detect urinary extravasation after RARP. However, it is not performed routinely at many centers around the world. Guru et al. reported that removal of catheter on postoperative days 8–10 without performing cystogram may not lead to complications in patients with RARP [26]. Although this prospective study was low-powered and consisted of only eighty patients recruited at a single institution, the fact remains that the fairly small percentage of men who develop complications relating to AUL may not be enough to justify the cost and manpower incurred by a routine cytogram for all patients with RARP. One prospective study consisting of 230 patients at a single institution indicated that selective cystogram is preferable in cases with bladder neck resection or a history of transurethral resection of the prostate [27]. Tillier et al. similarly suggested that selective cystogram in cases with larger prostate and with preoperative voiding problems may prevent early urinary retention and alleviate voiding complaints [9]. Based on these reports, a selective cystogram may be more beneficial than a routine cystogram. Prospective randomized studies under further standardization of cystogram protocols are required to better support this idea.

There are several limitations to discuss. First, this study was not a prospective randomized study, and further investigation is needed to confirm our assertion. Second, although our study analyzed the findings of a postoperative cystogram according to the criteria as defined by Williams et al., the degree of AUL would be influenced by the amount and speed of contrast instilled into the bladder, and hence cannot be said to completely adhere to the original report [8]. Third, we may underestimate the relationship between the surgical skill and the incidence of AUL since we only examined the total console time and not the actual time to complete UVA.

## Conclusion

In conclusion, we objectively examined the results of a routine cystogram after RARP and found that major AUL was associated with a longer time to recovery of continence. BMI was identified as the predictor of overall and major AUL and may be taken into consideration when performing selective cystograms after RARP.

## Data Availability

The datasets generated during the current study are not publicly available due to on-going clinical studies based on the database but are available from the corresponding author on reasonable request.

## Abbreviations

AUL: Anastomotic urinary leakage
BMI: Body mass index
GS: Gleason score
UVA: Urethrovesical anastomosis
LRP: Laparoscopic radical prostatectomy
PSA: Prostate specific antigen
PSM: Positive surgical margin
RARP: Robot-assisted radical prostatectomy
RRP: Retropubic radical prostatectomy
